# Icariin-conditioned serum engineered with hyaluronic acid promote repair of articular cartilage defects in rabbit knees

**DOI:** 10.1186/s12906-019-2570-0

**Published:** 2019-07-03

**Authors:** Juntao Zhang, Donglin Zhang, Chaochao Wu, Aifeng Liu, Chao Zhang, Jianjie Jiao, Man Shang

**Affiliations:** 1 Department of orthopedics, First Teaching Hospital of Tianjin University of Traditional Chinese Medicine, Tianjin, China; 2 Tianjin University of Traditional Chinese Medicine, Tianjin, China; 3 Department of pharmacology, School of Basic Medical Sciences, Tianjin Medical University, 22# Qixiangtai Road, Heping District, Tianjin, China

**Keywords:** Cartilage defect, Chondrocyte, Icarrin, Hyaluronic acid

## Abstract

**Background:**

Osteochondral defects mostly occur as a result of trauma or articular degeneration. The poor regenerative ability of articular cartilage remains osteochondral defects are a tricky problem to deal with. The modern treatment strategies mainly focus on cartilage tissue engineering with bioactive materials. In this study, we aimed to develop icariin conditioned serum (ICS) together with hyaluronic acid (HA) and determine their ability in reparing osteochondral tissue in a critical-sized defect in rabbit knees.

**Methods:**

Primary chondrocytes were incubated with serum conditioned with icariin at different concentrations, then cell proliferation rates and glycosaminoglycan (GAG) secretion were detected. Rabbits were treated with intra-articular injection of 0.5 mL normal saline (NS), ICS, HA and ICS + HA in the right knee joint, respectively. ICRS scores were used to assess the macroscopic cartilage regeneration. Histological and immunohistochemical analysis including H&E, Safranin O, toluidine blue and collagen II staining were used to determine the repair of cartilage and the regeneration of chondrocytes.

**Results:**

Icariin at a low dose of 0.94 g/kg was identified to have significantly promoted the proliferation of chondrocytes and enhance the secretion of GAG. Femoral condyle from rabbits treated by ICS together with HA was observed to be integrated with native cartilage and more subchondral bone regeneration. ICS together with HA could promote repair of the cartilage defect and increase the neoformation of cartilage.

**Conclusions:**

These results demonstrated the potential of ICS combined with HA to promote reparative response in cartilage defects and the possible application in bioactive material based cartilage regeneration therapies.

**Electronic supplementary material:**

The online version of this article (10.1186/s12906-019-2570-0) contains supplementary material, which is available to authorized users.

## Background

Articular cartilage is hyaline cartilage and located surrounding the articular surfaces of bones. Osteochondral defects mostly occur as a result of trauma, articular inflammation and degeneration. The biochemical breakdown and physical deterioration of articular cartilage results in joint disease and eventually leads to progressive total joint destruction. As articular cartilage is an avascular and aneural tissue by nature, the limited regenerative ability and poor self-healing capacity remains that joint injury or degenerative pathology like osteoarthritis are a thorny clinical problem to challenge.

Treatment strategies for osteochondral defects include nonpharmacological (e.g. physiotherapy), pharmacological (e.g. hormones) and intra-articular (e.g. injection of hyaluronic acid) therapies. The natural repair of osteochondral defects can be enhanced with biocompatible, biodegradable and bioactive materials that provide structural support and molecular cuing to stimulate repair. Current modalities like tissue engineering with combined materials and cell-based therapy are more and more widely used. Recently, autologous conditioned serum (ACS) has emerged as an alternative, safe and well-tolerated treatment in osteoarthritis (OA). Meijer et al. devised the biologic therapeutic preparation known as ACS, marketed as ‘Orthokinew’ (Orthogen, Düsseldorf, Germany), a medical device (a syringe) used to produce ACS enriched with anti-inflammatory cytokines [1]. Some potential responders considered to apply certain novel biological treatments such as ACS intra-articular injections for patients with a risk of osteoarthritis development [2]. Moreover, ACS is proposed to be capable of leading to enhancement of tissue regeneration and to reduction of degenerative mechanisms. Similarly, our previous research has prepared rabbit conditioned serum with a Chinese patent medicine “Xianlinggubao capsule”, and demonstrated the proliferation effects of conditioned serum on primary rabbit chondrocytes.

The *Epimedium* herb, a widely used traditional Chinese herbal medicine on arthritis in China, Japan and Korea, is the major ingredient of the Chinese patent capsule. Icariin (ICA) is a prenylated flavonol glycoside isolated from the *Epimedium* herb, and has been shown to be the main bioactive component. ICA has been reported to be more potent than other flavonoid compounds in promoting osteogenic differentiation and maturation of osteoblasts. Specifically, it has been reported that ICA is a safe and strong chondrocyte anabolic agent which can affect the proliferation of chondrocytes and reduce the degradation of the extracellular matrix (ECM) [3]. In addition, ICA could promote the expression of chondrogenesis genes of chondrocytes like aggrecan, collagen II and Sox9 genes [4]. Most interestingly, it has been reported that ICA can be delivered locally by biomaterials and that it has an osteoinductive effect on bone tissues. These researches have suggested that ICA is an effective accelerator for chondrogenesis and ICA-loaded biomaterials might have the potential for cartilage tissue engineering.

Currently, hyaluronic acid (HA) is widely used to treat osteoarthritis of the knee by intra-articular injection. It is a major constituent of synovial fluid. As an essential component in the ECM of cartilage, HA is involved in cell proliferation, morphogenesis, inflammation and wound repair. It has been demonstrated that HA gels combined with proteoglycan could serve as an injectable therapeutic agent for slowing or inhibiting the onset of OA after knee injury [5]. Furthermore, its combination with additional disease-modifying drugs could be an effective strategy since the gel can be used as a delivery vehicle for cartilage repair, especially in severe cartilage injury [6].

In the present study, we developed rabbit ICA conditioned serum (ICS) by gavaging rabbits with ICA. It was hypothesized that the biomaterial combining ICS together with HA could promote cartilage repair in an osteochondral defect model in vivo, and expectedly enhance the generation of proteoglycans and collagen.

## Methods

### Animals

New Zealand White rabbits were obtained from Academy of military medical science in Beijing. Rabbits (4 weeks) were used for isolation of primary chondrocytes, and adult rabbits (12 weeks) were used for drug conditioned serum (DCS) preparation and cartilage defect treatment. The rabbits were kept in special cages in a specific pathogen free (SPF) environment, allowing free movement and free access to food and drink. All procedures were performed in accordance with NIH guidelines for the Care and Use of Laboratory Animals (NIH Publications No. 80–23, revised 1996) and the research protocol was approved by Ethics Committee of Tianjin University of Traditional Chinese Medicine (TCM-LAEC20170026).

### Culture of primary chondrocytes

Primary chondrocytes were isolated from the articular cartilage of New Zealand White rabbits (four-week-old; Institute of military medical science; China). Briefly, the rabbits were sacrificed and cartilage tissue specimens were obtained from knee and shoulder joints. Fragments were tailored to approximately 1x1x1 mm, washed with cold phosphate buffer saline (PBS), and digested with 0.2% collagenase type II (Invitrogen, Carlsbab, CA, USA) for 4 h. Slices were then digested overnight at 37 °C with 0.025% collagenase type II in PBS on a magnetic stirrer. Isolated cells were suspended in dulbecco’s modified eagle medium (DMEM)/F-12 containing 10% fetal bovine serum (FBS) and 1% penicillin–streptomycin, cultured at 37 °C in a humidified atmosphere of 5% CO_2_. Chondrocytes corresponding to passage 3 or below were used for experiments.

### DCS preparation

Rabbits were treated with ICA (Lot: 160602, Shanghai ronghe, China) to prepare drug-conditioned serum for further administration on chondrocytes and cartilage defects. The equivalent dose of ICA on rabbits calculated according to humans is 0.47 g/kg. Twenty rabbits were randomly divided into four groups, including control, low, middle and high dose groups, which were administered with normal saline, ICA 0.94 g/kg, 2.35 g/kg, 4.70 g/kg at the volume of 10 mL/kg, respectively. Rabbits were gavaged with the different solutions everyday for 1 week. 2 h after the last administration, rabbits were anesthetized with 1 g/kg urethane and blood was sterilely drawn from common carotid artery. Then the blood was centrifuged at 2000 g for 10 min and the supernatants were filtered under 0.22 μm strainer. The DCS was prepared as freeze-dried powder by FD5–2.5 Freeze Dryer (SIM, Newark, USA) and could be freely soluble in normal saline or sodium hyaluronate injection.

### Cell proliferation assay

Cell proliferation rates were determined using a methyl thiazolyl tetrazolium (MTT, Amresco, Solon, OH, USA) method. Articular chondrocytes cultured in 96-well plates at 8 × 10^4^ cells/mL were treated with 10% DCS of different concentrations in DMEM/F12 for 72 h. Then culture supernatants were replaced with DMEM/F12 and incubated with 10 μL 0.5% MTT solution for 4 h at 37 °C. The absorbance of the blue formazan derivative was measured at a wavelength of 490 nm using a microplate reader (Bio-Rad Laboratories, CA, USA). The experiment was replicated independently for three times.

### Determination of glycosaminoglycan (GAG) contents

The amounts of GAG released from chondrocytes were measured using a 1, 9- dimethylmethylene blue (DMMB, Sigma) assay. Articular chondrocytes were plated in 24-well plates at 8 × 10^4^ cells/mL and treated with 10% DCS of different concentrations in DMEM/F12 for 72 h. The absorbance of cell lysates and chondroitin sulfate was detected at the wavelength of 525 nm by a microplate reader. The release of GAG contents were calculated according to the standard curve of chondroitin sulfate. The total DNA content was determined by a PicoGreen DNA kit (Invitrogen, Carlsbab, CA, USA) according to the manufacturer’s protocol and a fluorescence spectrophotometer (F-7000, Hiatachi, Japan) was used for the measurement. The contents of glycosaminoglycan (GAG) were normalized to the DNA content (μg GAG/μg DNA). Each experiment was replicated independently for three times.

### In vivo osteochondral defect model and treatment with HA and ICS

Twenty-four male New Zealand White rabbits of mean weight 2.9 kg (range 2.5–3.3 kg) were used for in vivo experiments. Animals were anesthetized by intramuscular injection of 30–40 mg/kg ketamine and fixed in supine position for surgery. A critical-sized defect, 4 mm diameter full-thickness osteochondral defect was created using a surgical drill on the load-bearing area medial femoral condyle to a depth of 3 mm [7]. Penicillin was intramuscularly injected to each rabbit at the dose of 40,000 U/d for 3 days following the operation. All animals were carefully evaluated during the first 24 h after operation.

Rabbits were randomly divided into four groups, including normal saline (NS), ICS (low dose freeze-dried DCS diluted by NS), HA and ICS + HA (low dose freeze-dried DCS diluted by HA) groups. At the beginning of the third week, rabbits in different groups were treated with intra-articular injection of 0.5 mL NS, ICS, HA and ICS + HA in the right knee joint, respectively. All animals were intra-articularly injected of normal saline in the left knee joint. No animals were observed to be infected throughout the experimental period. Rabbits were provided with individual cages and allowed to move freely after surgery. All rabbits were euthanized by an intravenous injection of 120 mg/kg of sodium pentobarbital (MTC Pharmaceuticals, Ontario, Canada) 12 weeks after surgery. The joints of each rabbits from all groups were harvested, and the reparative results were evaluated by macroscopic examination and histological analysis.

### In vivo cartilage defect repair evaluation

The repaired knee joints were retrieved from the hind limbs of the rabbits. The gross appearances of cartilage defects were photographed. The surface characteristics of the regenerated tissue and their integration with the surrounding tissue were blindly examined using the International Cartilage Repair Society (ICRS) macroscopic assessment scale as shown in Table 1 [8, 9].

**Table 1:**
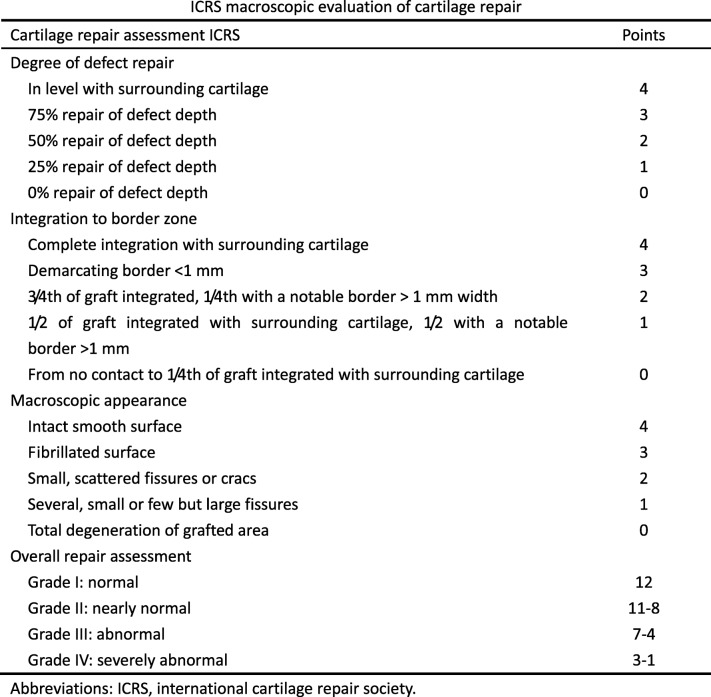
ICRS macroscopic evaluation of cartilage repair

### Histological and immunohistochemical analysis

For cultured chondrocytes, 2 × 10^4^ cells were seeded on slides per well of a 6-well plate and incubated for 2 days. Then chondrocytes were washed with PBS and fixed with 4% paraformaldehyde for 30 min. Toluidine blue staining were performed for characterization of chondrocytes. For characterization of collagen II expression of chondrocytes, cells were treated with HRP-conjugated anti-collagenII primary antibody (1/400 diluted, Bioss, Beijing, China) and visualized with a diaminobenzidine peroxidase substrate kit (Beyotime, Haimen, China). Stained cells were detected with an inverted fluorescent microscope (Ti-U, Nikon, Japan).

After gross examination, repaired knee joints were fixed, decalcified and dehydrated for further embedding. Hematoxylin and eosin (H&E) staining, toluidine blue and safranin O staining were performed for morphological evaluation. For determination of collagen II expression, tissue sections were treated the same with cells. Images were obtained using optical microscopy (YS-100; Nikon; Japan).

### Statistical analysis

Data was collected from parallel samples and presented in the manner of mean ± standard deviation (SD). All data was analyzed using ANOVA by SPSS 19.0 software. Differences between two treatments were assessed by student’s paired t-test. *P* values lower than 5% was considered statistically significant.

## Results

### Primary chondrocytes were characterized by histology staining

**Fig. 1:**
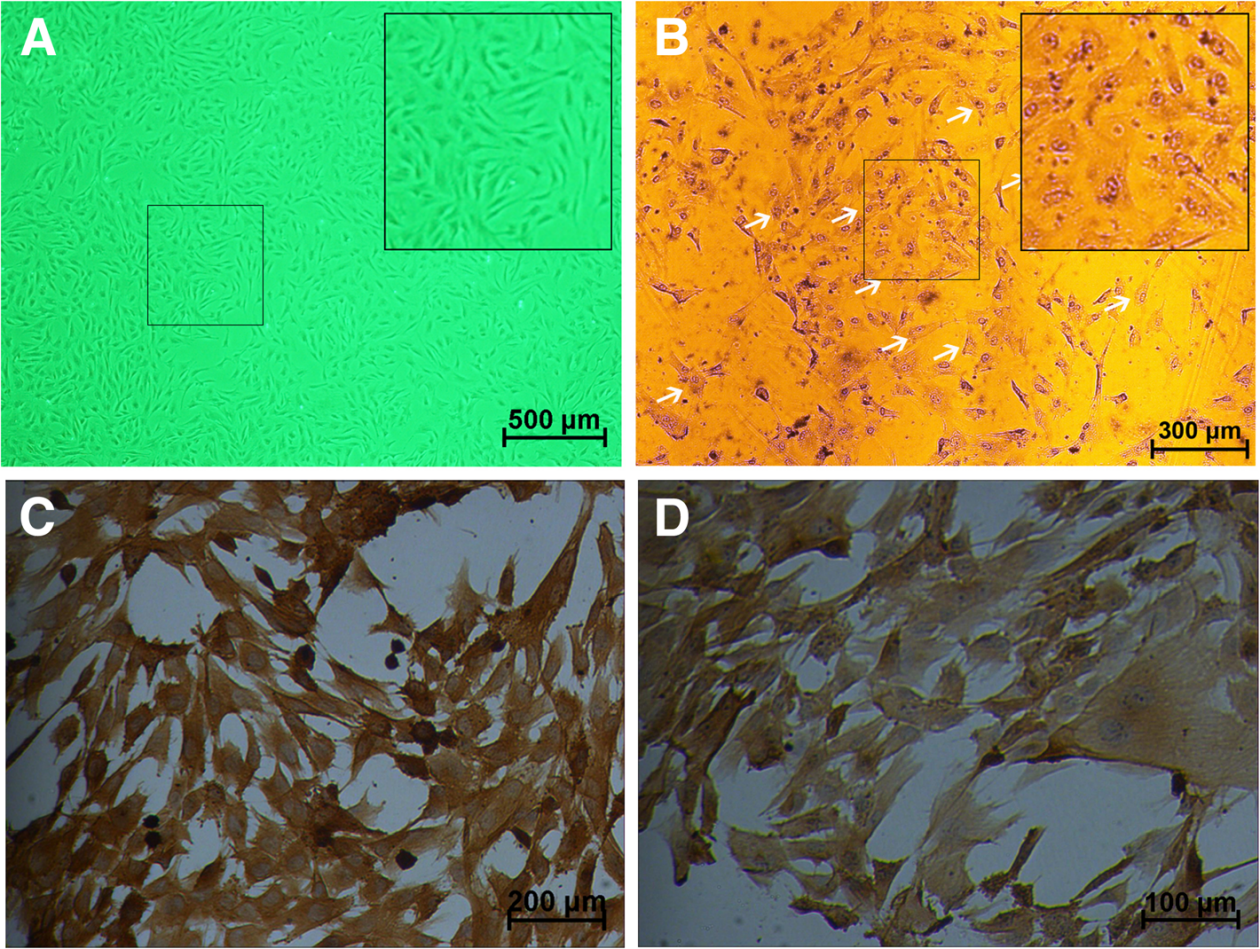
Characterization of primary chondrocytes. **a** Representative images of primary chondrocytes. **b** Toduiline blue staining of primary chondrocytes. **c**, **d** Immunohistological staining of collagen II for chondrocytes

Primary chondrocytes were isolated from knee and shoulder joints of rabbits and represented as polygonal or triangular shape with round or oval nucleus (Fig. 1a). At the same time, chondrocytes were characterized by toluidine blue staining and immunohistology of collagen II. The characteristic zonal structure of chondrocytes was embedded in matrix containing high amounts of proteoglycans. This was evidenced by toluidine blue staining (Fig. 1b). Further characteristic properties of chondrocytes are a strong collagen II staining, which was confirmed by positive tan - brown granulates in cytoplasma (Fig. 1c, d).

### DCS of low concentration could promote the proliferation of chondrocytes

**Fig. 2:**
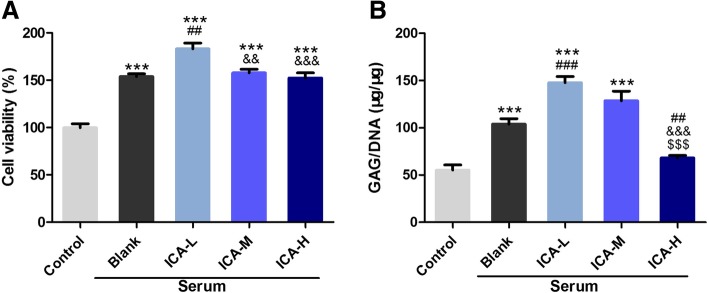
DCS of low concentration could promote the proliferation of chondrocytes. (*n* = 6, mean ± SD). **a** MTT assay of the proliferation rates of chondrocytes incubated with DCS of different concentrations. **b** GAG contents of chondrocytes treated by DCS of different concentrations. ^***^P < 0.001 vs Control; ^##^*P* < 0.01, ^###^*P* < 0.001 vs Blank Serum; ^&&^*P* < 0.01, ^&&&^*P* < 0.001 vs ICA-L, ^$$$^*P* < 0.001 vs ICA-M. Abbreviations: DCS, drug conditioned serum; GAG, glycosaminoglycan; MTT, methyl thiazolyl tetrazolium; SD, standard deviation. ICA-L refers to group treated with low dose icariin conditioned serum, ICA-M refers to group treated with middle dose icariin conditioned serum, ICA-H refers to group treated with high dose icariin conditioned serum

Chondrocytes were incubated with DCS of different concentrations and the proliferation rates were assessed by MTT assay. After being treated with DCS for 72 h, cells in serum groups exhibited strong proliferation capacity compared with control (*P* < 0.001, Fig. 2a). ICA-L (low dose icariin conditioned serum), ICA-M (middle dose icariin conditioned serum) and ICA-H (high dose icariin conditioned serum) groups showed much higher proliferation rates when compared with Blank-Serum group (*P* < 0.001, Fig. 2a). Specifically, chondrocytes in ICA-L group presented strongest growth ability (*P* < 0.001, Fig. 2a). The GAG is one of main components of cartilage extracellular matrix and the GAG/DNA ratio can be considered as the marker of chondrogenic proliferation. Cells in Blank-Serum, ICA-L and ICA-M groups showed more GAG secretion compared with control (P<0.001, Fig. 2b). In the similar manner with cell proliferation rates, chondrocytes in ICA-L group have the most amount of GAG production (*P* < 0.001, Fig. 2b). Therefore, DCS prepared with ICA of low concentration was applied in the further in vivo experiment.

### In vivo cartilage defect regeneration

**Fig. 3:**
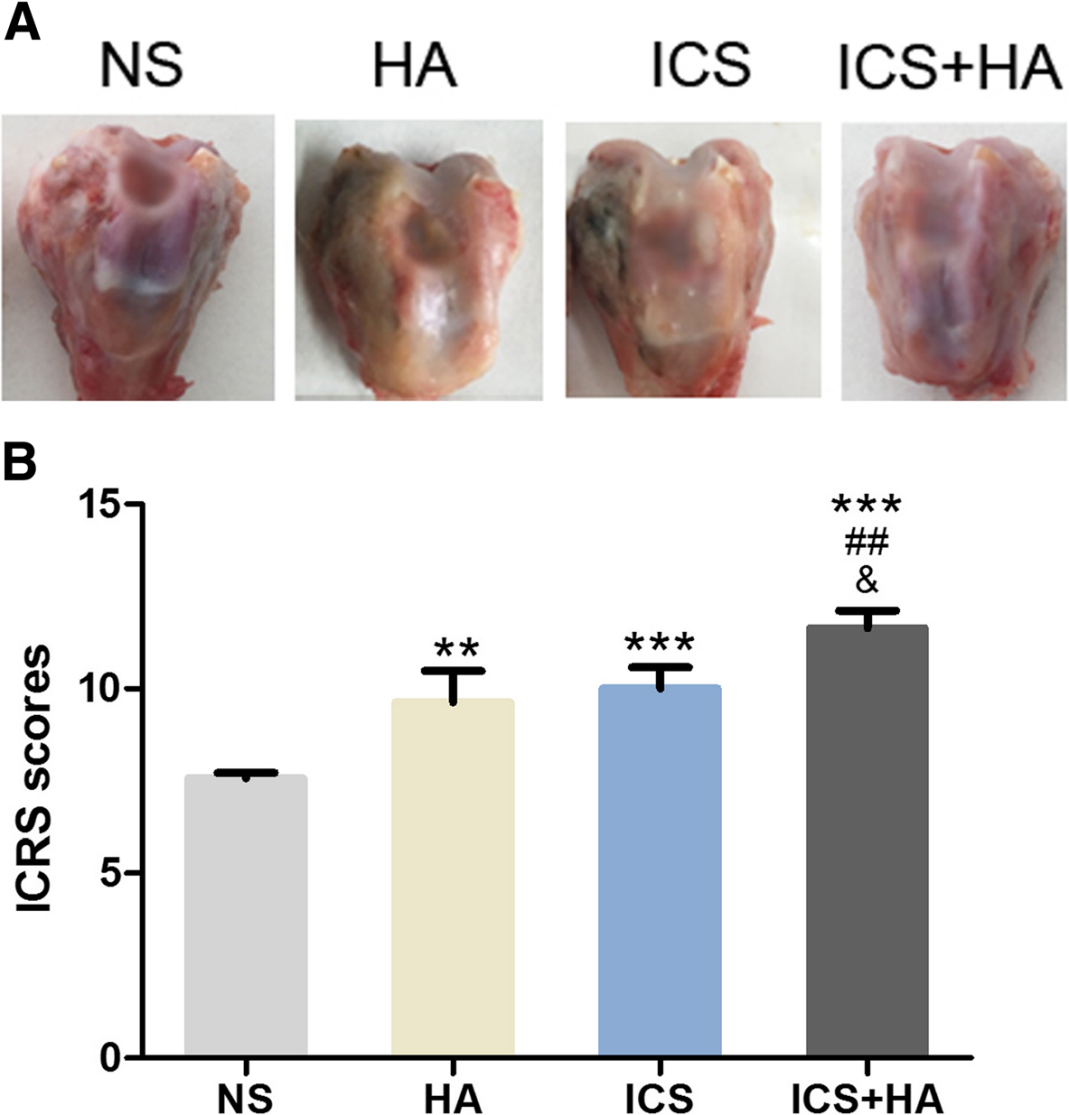
Gross observation and evaluation of the repair of osteochondral defects in rabbit knees. **a** Gross observation of the repair of osteochondral defect. **b** Repair of osteochondral defects assessed by ICRS scores. (*n* = 6, mean ± SD). Abbreviations: ICRS, international cartilage repair society; SD, standard deviation

HA is a widely used drug for the treatment of osteoarthritis of the knee. As DCS of ICA-L were confirmed to be effective on the proliferation of chondrocytes in vitro, it was applied for the treatment of rabbit articular cartilage defect together with HA. After surgery for 12 weeks, the knee joints of each rabbits were harvested and evaluated by gross morphology examination, histological and immunohistochemical evaluation, as well as semi-quantitative histological scoring analysis. The general morphology observation revealed that the defects were incompletely filled with uneven neo-tissue in 12 weeks in NS group. And the surface of femoral condyle cartilage was not intact, with the exposure of subchondral bone. However, the defect in ICS + HA group was almost repaired and the cartilage surface looked milky and smooth without obvious adhesion (Fig. 3a). The poor degradation of cartilage also exhibited good recovery of femoral condyle. In addition, the defect area was analyzed histologically using the ICRS classification of cartilage regeneration. The ICRS macroscopic scores revealed that the treatment groups acquired significantly high scores when compared with NS group (*P* < 0.05, Fig. 3b). Among those, ICS + HA group showed much better repair of cartilage defect than other treatment groups (*P* < 0.05, Fig. 3b). The higher ICRS scores in ICS + HA group indicated better recovery of cartilage defect.

**Fig. 4:**
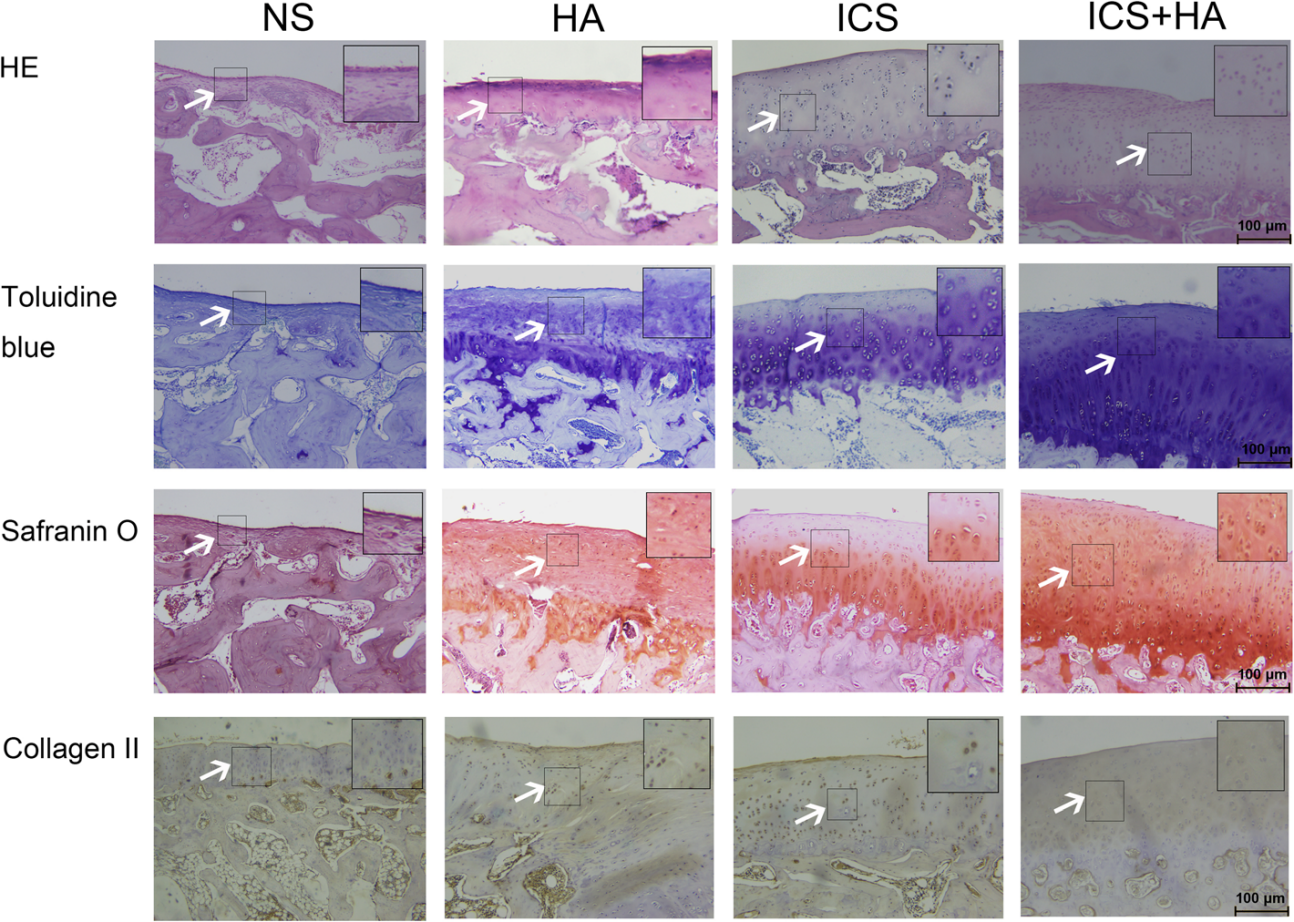
Histological and immunohistochemical analysis of osteochondral defects repair in rabbit knees. Arrows pointed out the significant changes of regenerated tissues in the four groups

After 12 weeks of healing, H&E staining were performed on the articular joint samples to assess subchondral bone regeneration. In NS group, the regenerated tissues were thinner than treatment groups, with very few hyaline cartilage cells, suggesting inferior tissue integration. However, ICS group and HA group showed more regenerated cartilage than NS group. The repaired tissues in ICS + HA group were totally integrated with surrounding native cartilage. Toluidine blue staining for chondrocytes showed more hyaline cartilage, but few fibrous cartilage in ICS + HA group than other groups. Moreover, the strong Safranin-O staining for GAG, immunehistochemical staining for collagen II also demonstrated that the newly formed tissue was predominantly hyaline cartilage, the same as native cartilage (Fig. 4). The above results confirmed that ICS combined with HA could significantly promote the cartilage defect repair and increase the neoformation of cartilage.

## Discussion

Following trauma or degenerative pathology, native osteochondral repair is often inadequate due to the inherent properties of the cartilage. Recently, drug-loaded materials have been proposed as a potential effective strategy for the repair of osteochondral defect. In the present study, it was demonstrated that serum conditioned with low dose ICA could highly promote the proliferation of cultured primary chondrocytes and enhance the production of GAG. ICA is the major bioactive ingredients of the Epimedium herb that can induce the proliferation of chondrocytes and promote the process of chondrogenic ECM formation. GAG, the main ECM of cartilage, can be secreted from chondrocytes and represent the growth condition of chondrocytes. It was observed that GAG secretion in cultured chondrocytes was enhanced by supplementation of serum, especially the serum conditioned with low dose ICA. Thus, ICS was considered as drug-containing biological agents for use in cartilage repair and regeneration.

The overall aim of this study was to assess the in vivo response of ICS combined with HA and determine its potential to facilitate the repair of osteochondral tissue in a critical-sized defect in a rabbit knee. Specifically, we investigated the effects of ICS combined with HA on the repair of cartilage defect. The ICRS score showed that the combination of ICS and HA could enhance the macroscopic morphological appearance of the articular surface compared to empty defect controls, as well as histological evaluation. The ICS + HA group showed better subchondral bone regeneration, more hyaline cartilage, strong expression of GAG and collagen II.

It has been stated that HA gels with proteoglycan could alleviate the onset of OA after knee injury, and in situ cross-linkable HA hydrogel could improve cartilage regeneration [10, 11]. Solchaga et al. showed the fibronectin-coated hyaluronan-based scaffold could organize the natural response and facilitate the integration of the neo-cartilage with the adjacent tissue [12].In our study, HA could partially restore chondral defects and favor chondrogenesis. We also applied ICS to promote and provide molecular cuing to stimulate repair. ICS could increase the synthesis of GAG and collagen type II, and accelerate the formation of chondroid tissue in the defect area. It even improved the restoration efficiency of the critical-sized osteochondral defects in adult rabbit model and enhanced the integration of new-formed cartilage with subchondral bone. ICS was prepared by gavaging rabbits with icariin at the dose of 0.94 g/kg.

Metabolic and pharmacokinetic studies have shown that icariin can be metabolized by intestinal flora and converted to its derivatives like icaritin, icariside I, icariside II, and desmethylicaritin [13, 14]. Studies have provided evidence of the broad therapeutic capabilities of icariin, especially with reference to its osteoprotective effects [15]. Icariin can be a potential promoting compound for cartilage tissue engineering and a substitute for the use of some growth factors. Therefore, we attempted to use the serum pharmacology method to explore its effects on cartilage tissue engineering.

Some prospective randomized controlled trials have considered ACS as an interesting, well-tolerated and possibly effective option in human knee OA [16]. The ACS production process has been shown to reproducibly elevate IL-1Ra and other factors [17, 18]. It’s a definite possibility that the mechanism of ACS may be that the therapeutic molecules help to re-establish a healthy joint homeostasis [19]. In the present study, we have modified the rabbit serum conditioned with traditional Chinese medicine ingredients icariin. Indeed, in the rabbit osteochondral defect model, we found that ICS combined with HA significantly enhanced articular cartilage repair compared with controls which could be indexed by improved ICRS histological score of the repair region. It is interesting to note that Safranin O staining of cartilage area in ICS + HA group is increased accompanied by enhanced toluidine blue and collagen II staining of subchondral bone formation.

However, the mechanisms by which the effects are mediated by ICS and HA are not fully understood. An important limitation of ICS is needed to explore the actual composition and the effective components of it. Two human trials have evaluated the efficacy of ACS marketed as Orthokine in symptomatic OA of the knee. Baltzer et al. has tested ACS was superior to saline and hyaluronan (HA) as an intra-articular therapy to reduce the signs and symptoms of knee OA. However, the direct effect of the entire blend of known and unknown factors present in ACS on the metabolism of articular cartilage in human OA has not been described. Only limited data is available on the actual composition of the conditioned serum. ICA and its derivatives in the serum might behave as growth factors and participate in the osteochondral repair process.

## Conclusions

ICA or HA may partially exert positive effects on promoting the integration of the superficial layer of cartilage and subchondral bone, while the accurate mediation of the process by ICA and HA needs to be further investigated. Taken together, our results suggested that ICS combined with HA could promote articular cartilage repair through coordinating chondrocytes proliferation and integration with subchondral bone formation.

## Additional files

Additional file 1: **Table S1.** Raw data for Fig 2. A. Proliferation rates of chondrocytes treated with DCS of different concentrations. B. GAG contents of chondrocytes treated by DCS of different concentrations. (DOC 46 kb)

Additional file 2: **Table S2.** Raw data for Fig 3b. Repair of osteochondral defects assessed by ICRS scores. (DOC 35 kb)

Additional file 3: **Table S3.** Weights of rabbtits. Baseline data for animals used for cartilage defect experiment. (DOC 32 kb)

Additional file 4: **Figure S1.** Histological evaluation methods of articular cartilage (Mankin scoring system). Mankin histological scores (DOC 156 kb)

Additional file 5: **Figure S2.** Proliferation rates. Proliferation rates of chondrocytes treated with DCS of different concentrations. (DOC 42 kb)

Additional file 6: Flow charts. Flow chart1, 2. The timeline of what happened to each group of rabbits. (DOCX 74 kb)

## Data Availability

The datasets analyzed during the current study are available from the corresponding author on reasonable request. All data supporting the conclusions are included in the article and Additional file 1 Table S1, Additional file 2: Table S2, Additional file 3: Table S3, Additional file 4: Figure S1, Additional file 5: Figure S2, and Additional file 6: Flow charts.

## Abbreviations

ACS: Autologous conditioned serum
DCS: Drug conditioned serum
DMEM: Dulbecco’s modified eagle medium
DMMB: Dimethylmethylene blue
ECM: Extracellularmatrix
FBS: Fetal bovine serum
GAG: Glycosaminoglycan
H&E: Hematoxylin & eosin
HA: Hyaluronic acid
ICA: Icariin
ICRS: International cartilage repair society
ICS: Icariin conditioned serum
MTT: Methyl thiazolyl tetrazolium
NS: Normal saline
OA: Osteoarthritis
PBS: Phosphate buffer saline
SD: Standard deviation
SPF: Specific pathogen free
