# Correction : Icariin-conditioned serum engineered with hyaluronic acid promote repair of articular cartilage defects in rabbit knees

**DOI:** 10.1186/s12906-023-04133-2

**Published:** 2023-08-24

**Authors:** Juntao Zhang, Donglin Zhang, Chaochao Wu, Aifeng Liu, Chao Zhang, Jianjie Jiao, Man Shang

**Affiliations:** 1 Department of orthopedics, First Teaching Hospital of Tianjin University of Traditional Chinese Medicine, Tianjin, China; 2 Tianjin University of Traditional Chinese Medicine, Tianjin, China; 3 Department of pharmacology, School of Basic Medical Sciences, Tianjin Medical University, 22# Qixiangtai Road, Heping District, Tianjin, China

**Correction: BMC Complement Altern Med 19, 155 (2019)**

10.1186/s12906-019-2570-0

Following publication of the original article [1], the authors identified an error in Fig. 4. The correct figure is given below.

**Fig. 4:**
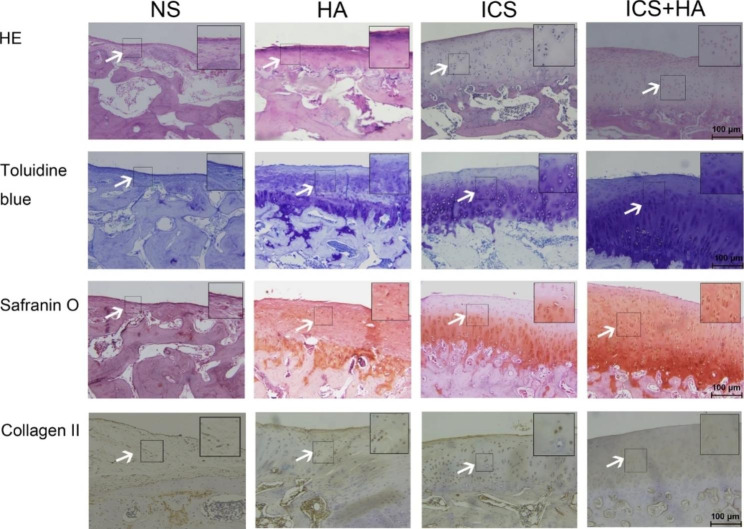
Histological and immunohistochemical analysis of osteochondral defects repair in rabbit knees. Arrows pointed out the significant changes of regenerated tissues in the four groups

The original article has been corrected.
