# Supplementary material for: Icariin-conditioned serum engineered with hyaluronic acid promote repair of articular cartilage defects in rabbit knees
Source: BMC Complement Altern Med. 2019 Jul 3;19:155. doi: 10.1186/s12906-019-2570-0 (PMC6610878; doi:10.1186/s12906-019-2570-0)
Supplement: Supplementary file 1 — Table S1. Raw data for Fig 2. A. Proliferation rates of chondrocytes treated with DCS of different concentrations. B. GAG contents of chondrocytes treated by DCS of different concentrations. (DOC 46 kb) [file 12906_2019_2570_MOESM1_ESM.doc]

**Table S1.** Raw data for Fig 2.

**A. Proliferation rates of chondrocytes treated with DCS of different concentrations.**

| Group | Experiment | | | | | | Mean±SD |
| --- | --- | --- | --- | --- | --- | --- | --- |
|  | 1 | 2 | 3 | 4 | 5 | 6 |  |
| Control | 116.0 | 103.8 | 97.8 | 100.4 | 95.7 | 84.7 | 99.7±10.3 |
| Blank | 147.2 | 162.6 | 150.0 | 158.3 | 160.0 | 144.2 | 153.7±7.6 |
| ICA-L | 164.6 | 176.3 | 204.3 | 178.1 | 177.0 | 198.0 | 183.1±15 |
| ICA-M | 156.6 | 149.8 | 169.3 | 149.4 | 152.7 | 169.0 | 157.8±9.2 |
| ICA-H | 147.4 | 159.6 | 176.2 | 146.4 | 143.3 | 140.3 | 152.2±13.5 |

Abbreviations: DCS, drug conditioned serum; SD, standard deviation. ICA-L refers to group treated with low dose icariin conditioned serum, ICA-M refers to group treated with middle dose icariin conditioned serum, ICA-H refers to group treated with high dose icariin conditioned serum.

**B. GAG contents of chondrocytes treated by DCS of different concentrations.**

| Group | Experiment | | | | | | Mean±SD |
| --- | --- | --- | --- | --- | --- | --- | --- |
|  | 1 | 2 | 3 | 4 | 5 | 6 |  |
| Control | 78.1 | 43.3 | 65.2 | 50.1 | 48.0 | 48.1 | 55.5±13.4 |
| Blank | 123.6 | 88.0 | 113.0 | 108.2 | 103.1 | 88.4 | 104.1±14 |
| ICA-L | 150.4 | 143.6 | 138.1 | 133.2 | 178.7 | 145.8 | 148.3±16.1 |
| ICA-M | 130.3 | 125.7 | 105.4 | 150.5 | 163.0 | 98.1 | 128.8±25.1 |
| ICA-H | 73.2 | 58.4 | 70.1 | 70.7 | 75.2 | 63.5 | 68.5±6.3 |

Abbreviations: DCS, drug conditioned serum; GAG, glycosaminoglycan; SD, standard deviation. ICA-L refers to group treated with low dose icariin conditioned serum, ICA-M refers to group treated with middle dose icariin conditioned serum, ICA-H refers to group treated with high dose icariin conditioned serum.
