# Supplementary material for: Icariin-conditioned serum engineered with hyaluronic acid promote repair of articular cartilage defects in rabbit knees
Source: BMC Complement Altern Med. 2019 Jul 3;19:155. doi: 10.1186/s12906-019-2570-0 (PMC6610878; doi:10.1186/s12906-019-2570-0)
Supplement: Supplementary file 2 — Table S2. Raw data for Fig 3b. Repair of osteochondral defects assessed by ICRS scores. (DOC 35 kb) [file 12906_2019_2570_MOESM2_ESM.doc]

**Table S2.** Raw data for Fig 3B.

**Repair of osteochondral defects assessed by ICRS scores.**

| Group | Experiments | | | | Mean±SD |
| --- | --- | --- | --- | --- | --- |
|  | 1 | 2 | 3 | 4 |  |
| NS | 7.5 | 7.5 | 7.8 | 7.5 | 7.6±0.1 |
| HA | 10.5 | 10.5 | 9.5 | 9.5 | 10±0.6 |
| ICS | 10 | 9.5 | 10.5 | 8.5 | 9.6±0.9 |
| ICS+HA | 11.5 | 12 | 11 | 12 | 11.6±0.5 |

Abbreviations: ICRS, international cartilage repair society; SD, standard deviation; HA, hyaluronic acid; ICA, icariin; NS, normal saline.
