# Supplementary material for: Icariin-conditioned serum engineered with hyaluronic acid promote repair of articular cartilage defects in rabbit knees
Source: BMC Complement Altern Med. 2019 Jul 3;19:155. doi: 10.1186/s12906-019-2570-0 (PMC6610878; doi:10.1186/s12906-019-2570-0)
Supplement: Supplementary file 3 — Table S3. Weights of rabbtits. Baseline data for animals used for cartilage defect experiment. (DOC 32 kb) [file 12906_2019_2570_MOESM3_ESM.doc]

**Table S3.**

**Baseline data for animals used for cartilage defect experiment.**

| Group | Weight (kg) | microbiological status |
| --- | --- | --- |
| NS | 2.9±0.3 | Specific pathogen free |
| HA | 3.0±0.3 | Specific pathogen free |
| ICS | 3.0±0.2 | Specific pathogen free |
| ICS+HA | 2.9±0.2 | Specific pathogen free |

Abbreviations: HA, hyaluronic acid; ICA, icariin; NS, normal saline.
