# Supplementary material for: Icariin-conditioned serum engineered with hyaluronic acid promote repair of articular cartilage defects in rabbit knees
Source: BMC Complement Altern Med. 2019 Jul 3;19:155. doi: 10.1186/s12906-019-2570-0 (PMC6610878; doi:10.1186/s12906-019-2570-0)
Supplement: Supplementary file 4 — Figure S1. Histological evaluation methods of articular cartilage (Mankin scoring system). Mankin histological scores (DOC 156 kb) [file 12906_2019_2570_MOESM4_ESM.doc]

**Figure S1**

**
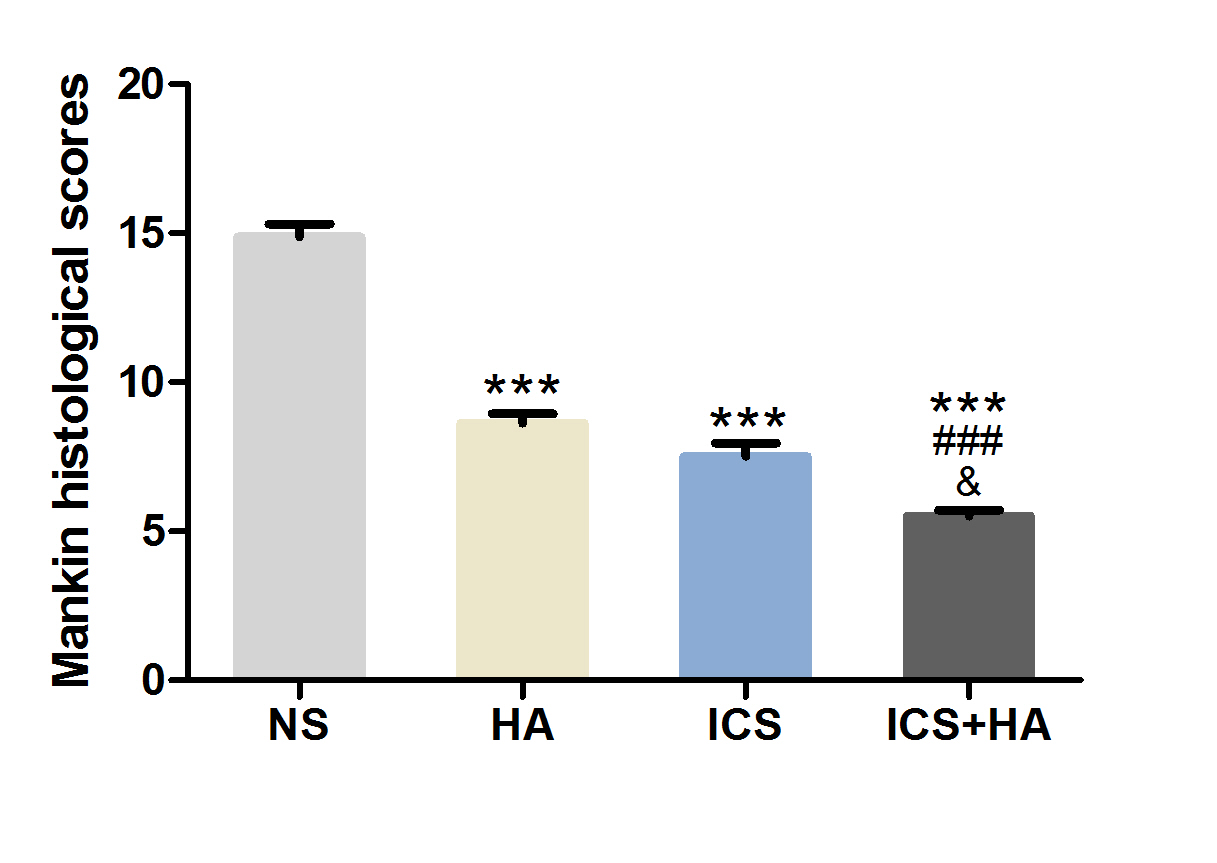
**

Histological evaluation methods of articular cartilage (Mankin scoring system) (n=6, mean±SD).

Abbreviations: ICRS, international cartilage repair society; SD, standard deviation; HA, hyaluronic acid; ICA, icariin; NS, normal saline.

**Table S4 Histological evaluation methods of articular cartilage (Mankin scoring system)**

| Articular cartilage |
| --- |
| (1) Structure (7 grades) |
| (2) Cell (4 grades) |
| (3) Safranin-O staining (5 grades) |
| (4) Tidemark (2 grades) |

Reference: Mankin HJ, Dorfman H, Lippiello L, et al. Biochemical and metabolic abnormalities in articular cartilage from osteoarthritic human hips. II. Correlation of morphology with biochemical and metabolic data. J Bone Joint Surg [Am] 1971;53-A:523-531.
