# Supplementary material for: Icariin-conditioned serum engineered with hyaluronic acid promote repair of articular cartilage defects in rabbit knees
Source: BMC Complement Altern Med. 2019 Jul 3;19:155. doi: 10.1186/s12906-019-2570-0 (PMC6610878; doi:10.1186/s12906-019-2570-0)
Supplement: Supplementary file 5 — Figure S2. Proliferation rates. Proliferation rates of chondrocytes treated with DCS of different concentrations. (DOC 42 kb) [file 12906_2019_2570_MOESM5_ESM.doc]

**Figure S2.**

**
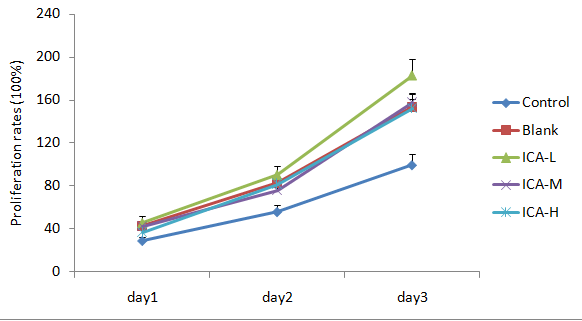
**

Proliferation rates of chondrocytes treated with DCS of different concentrations.

(n=6, mean±SD)

Abbreviations: DCS, drug conditioned serum; SD, standard deviation. ICA-L refers to group treated with low dose icariin conditioned serum, ICA-M refers to group treated with middle dose icariin conditioned serum, ICA-H refers to group treated with high dose icariin conditioned serum.
