# Supplementary figures and images for: Icariin-conditioned serum engineered with hyaluronic acid promote repair of articular cartilage defects in rabbit knees
Source: BMC Complement Altern Med. 2019 Jul 3;19:155. doi: 10.1186/s12906-019-2570-0 (PMC6610878; doi:10.1186/s12906-019-2570-0)

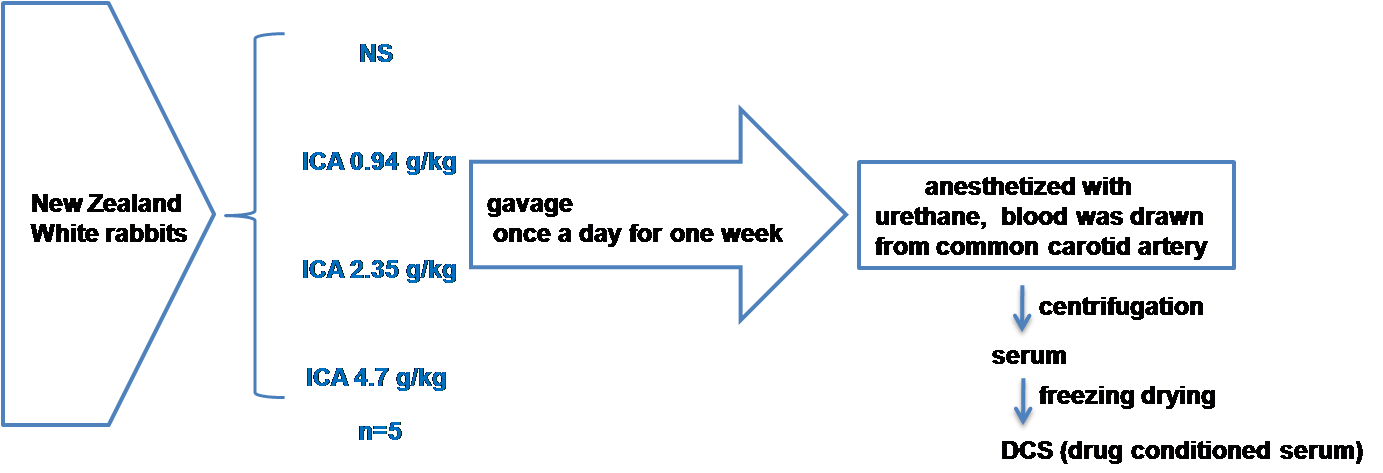


**Flow chart for DCS preparation**

**
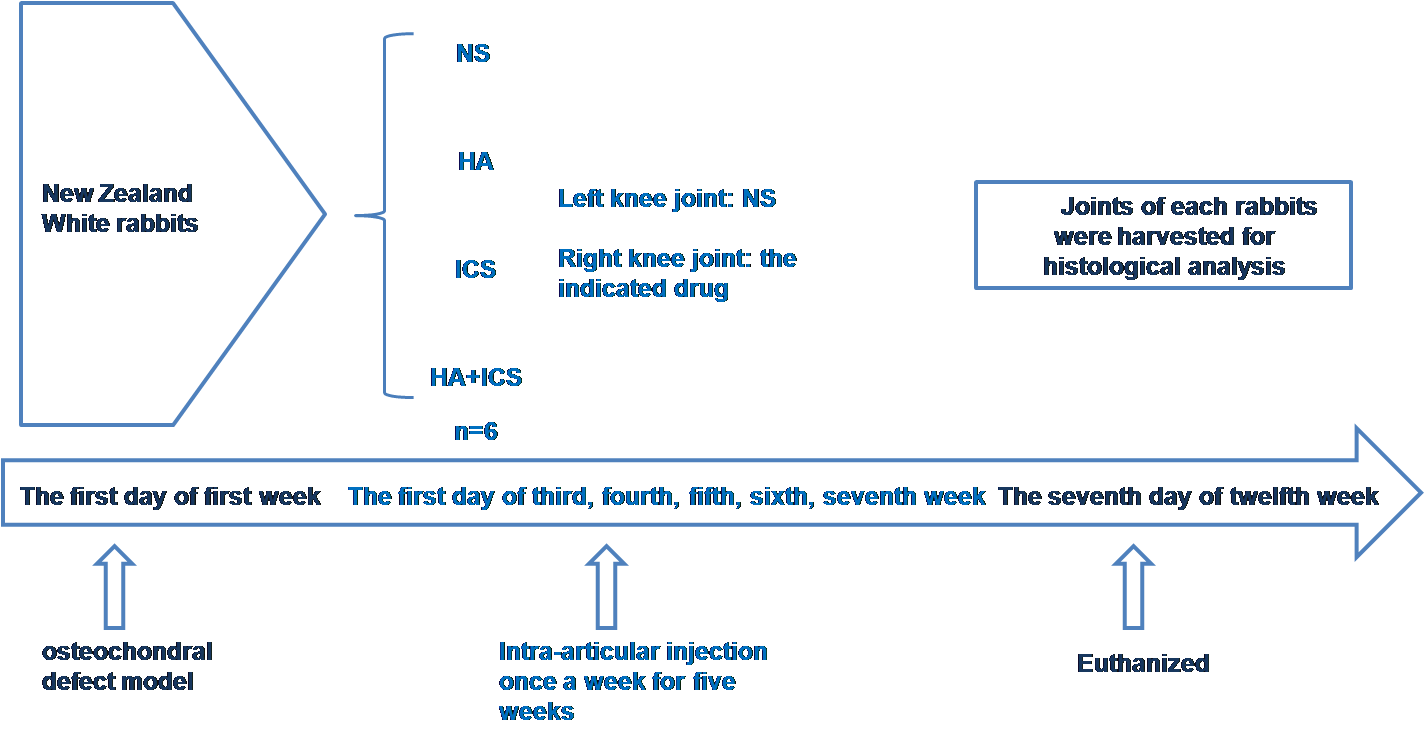
**

**Flow chart for osteochondral defect experiment *in vivo***

Supplement: Supplementary file 6 — Flow charts. Flow chart1, 2. The timeline of what happened to each group of rabbits. (DOCX 74 kb) [file 12906_2019_2570_MOESM6_ESM.docx]
